# The Complement Pathway: New Insights into Immunometabolic Signaling in Diabetic Kidney Disease

**DOI:** 10.1089/ars.2021.0125

**Published:** 2022-10-05

**Authors:** Sih Min Tan, Matthew Snelson, Jakob A. Østergaard, Melinda T. Coughlan

**Affiliations:** ^1^ Department of Diabetes, Central Clinical School, Alfred Medical Research and Education Precinct, Monash University, Melbourne, Australia.; ^2^ Department of Endocrinology and Internal Medicine, Aarhus University Hospital, Aarhus, Denmark.; ^3^ Steno Diabetes Center Aarhus, Aarhus University Hospital, Aarhus, Denmark.; ^4^ Baker Heart & Diabetes Institute, Melbourne, Australia.

**Keywords:** complement, kidney, diabetes, immunometabolism

## Abstract

**Significance:**

The metabolic disorder, diabetes mellitus, results in microvascular complications, including diabetic kidney disease (DKD), which is partly believe to involve disrupted energy generation in the kidney, leading to injury that is characterized by inflammation and fibrosis. An increasing body of evidence indicates that the innate immune complement system is involved in the pathogenesis of DKD; however, the precise mechanisms remain unclear.

**Recent Advances:**

Complement, traditionally thought of as the prime line of defense against microbial intrusion, has recently been recognized to regulate immunometabolism. Studies have shown that the complement activation products, Complement C5a and C3a, which are potent pro-inflammatory mediators, can mediate an array of metabolic responses in the kidney in the diabetic setting, including altered fuel utilization, disrupted mitochondrial respiratory function, and reactive oxygen species generation. In diabetes, the lectin pathway is activated via autoreactivity toward altered self-surfaces known as danger-associated molecular patterns, or via sensing altered carbohydrate and acetylation signatures. In addition, endogenous complement inhibitors can be glycated, whereas diet-derived glycated proteins can themselves promote complement activation, worsening DKD, and lending support for environmental influences as an additional avenue for propagating complement-induced inflammation and kidney injury.

**Critical Issues:**

Recent evidence indicates that conventional renoprotective agents used in DKD do not target the complement, leaving this web of inflammatory stimuli intact.

**Future Directions:**

Future studies should focus on the development of novel pharmacological agents that target the complement pathway to alleviate inflammation, oxidative stress, and kidney fibrosis, thereby reducing the burden of microvascular diseases in diabetes. *Antioxid. Redox Signal*. 37, 781–801.

## Introduction

Diabetes is the most common cause of end stage renal disease (ESRD) worldwide, and almost 40% of people living with diabetes will develop diabetic kidney disease (DKD) (5). Individuals with DKD carry a higher risk of developing comorbidities such as cardiovascular disease and, indeed, renal dysfunction is the major predictor of all-cause mortality in both type 1 diabetes (T1D) and type 2 diabetes (T2D) (3, 51).

Intensive glucose control after the onset of complications does not reduce the risk of DKD progression or improved overall clinical outcomes (5). Further, anti-hypertensive therapies, although reducing cardiovascular and all-cause mortality risk, do not reduce end stage kidney disease (28, 127). Sodium glucose cotransporter 2 (SGLT2) inhibitors are a new class of oral anti-hyperglycemic medications that are approved and indicated for T2D and have shown promising renoprotective effects independent of their glucose-lowering action (50). However, their use in T1D is limited, mainly due to safety concerns such as diabetic ketoacidosis (162). Therefore, there is still a critical need to identify pathogenic factors responsible for the onset and progression of DKD to find new therapeutic targets.

## Diabetic Kidney Disease

Classically, stages of DKD include glomerular hyperfiltration, which progresses to persistent albuminuria associated with hypertension and declining glomerular filtration rate (GFR), and ultimately ESRD, which requires dialysis or kidney transplant. Metabolic changes associated with diabetes, including hyperglycemia, hyperlipidemia, and insulin resistance, lead to glomerular hypertrophy, inflammation, and renal injury such as glomerulosclerosis and tubulointerstitial fibrosis (5).

The pathogenesis of DKD is complex and poorly understood. Over the years, studies have identified many molecular mechanisms of DKD, including, but not limited to, oxidative stress (110), enhanced flux into the polyol and hexosamine pathways (35, 130), activation of protein kinase C (PKC) and transforming growth factor-β (TGF-β) signaling pathways (22), increased advanced glycation endproduct (AGE) formation (146), epigenetic changes (145), impaired autophagy (32), and mitochondrial dysfunction (43).

Of those, oxidative stress has gained the most attention. It was believed that increased oxidative stress is the common pathway linking diverse mechanisms for the pathogenesis of diabetic complications (15). Brownlee subsequently put forward the hypothesis that a single unifying mechanism for the pathogenesis of diabetic complications is the overproduction of superoxide by the mitochondrial electron transport chain (19).

Since then, this widely held dogma is being challenged as more is known of the dual nature and complex role of reactive oxygen species (ROS) in different cell types in the context of diabetes (26). Nonetheless, the complex interplay between these activation pathways as well as downstream signaling has impeded the success of developing one single effective cure for DKD. Thus, a better understanding of each molecular mechanisms of DKD is crucial for the identification of novel targets for future drug therapy.

Although DKD is not traditionally viewed as an immune-modulated disease, immune cells and resident renal cells that activate innate immunity have been shown to be critical mediators of DKD (41, 62, 142). The complement system is one of the major effector mechanisms of the innate immune system that facilitates pathogen clearance via lysis, inflammation, and opsonization. However, the complement system also causes kidney injury in a variety of diseases, which has been extensively reviewed elsewhere (100, 147, 155). This review will summarize the findings from preclinical and clinical studies that show the complement cascade as an important mediator of DKD and provide insight into the newly discovered mechanisms by which the complement mediates metabolic processes and renal injury in DKD.

## Complement and DKD

The complement cascade can be activated through three main pathways: the classical, the mannose-binding lectin (MBL), and the alternative pathway (41). The classical pathway is activated by the binding of immune complexes such as IgG and IgM to C1q. Activation of the MBL pathway is triggered by microbial cell surface carbohydrates facilitated by MBL or other pattern recognition molecules (PRM) such as ficolins.

**FIG. 1.**
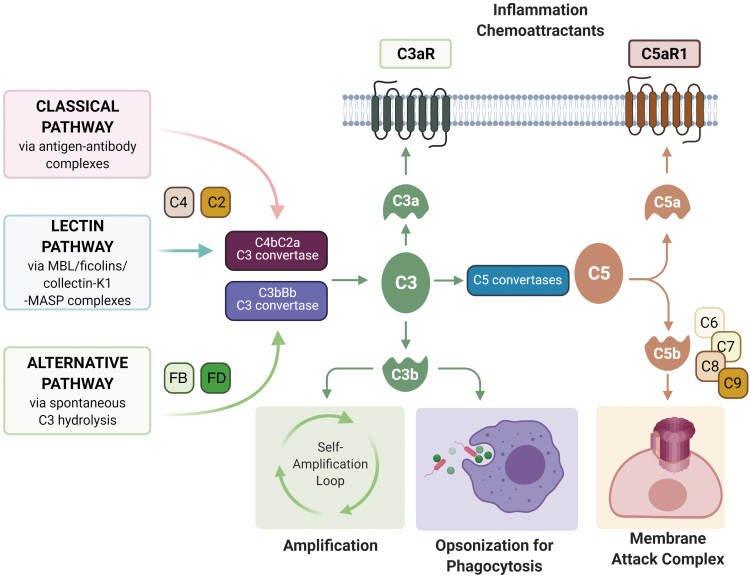
**The complement cascade.** The complement cascade can be activated via the classical (immune complexes), lectin (ficolins/collectin-K1/MBL), and alternative (spontaneous C3 hydrolysis) pathways. The classical and lectin pathways generate C4bC2a C3 convertase whereas the alternative pathway forms C3bBb C3 convertase, both of which cleave C3 molecules to form C3a and C3b. C3b can interact with factor B (FB), facilitating the cleavage of factor B into Ba and Bb by factor D (FD) to continuously cleave C3 molecules in a self-amplification loop. C3 convertases form multimeric complexes with additional C3b molecules to form C5 convertases, C3b_2_Bb to cleave C5 to C5a and C5b. C5b then binds to C6, C7, C8, and C9 to form C5b-9, the MAC. C3b acts as an opsonin that leads to the phagocytosis of invading microbes. The anaphylatoxins C3a and C5a are the main effector molecules of the complement cascades and exert their effector functions through binding and activation of their G-protein-coupled receptors, C3aR and C5aR1, resulting in the release of pro-inflammatory and chemoattractant signals. MAC, membrane attack complex; MBL, mannose-binding lectin. Color images are available online.

In the alternative pathway, complement is continuously activated at a tick-over rate and is further triggered by contact with various proteins, lipids, and carbohydrate structures on microbial and other foreign surfaces (Fig. 1).

Each of these pathways results in the formation of C3 convertases (C4b2a for classical and lectin pathways, C3bBb for alternative pathway) that continuously cleave C3 molecules, forming C3a and C3b, the latter of which can interact with factor B, facilitated by factor D to form more C3 convertase, making C3 cleavage the central amplification step of the cascade. C3 convertases then form multimeric complexes with additional C3b molecules to form C5 convertases, C4b2a3b and C3b_2_Bb, which cleave C5 to C5a and C5b.

C5b then binds to C6 and subsequently C7, C8, and C9, forming the C5b-9 membrane attack complex (MAC). MAC punches a pore in the membrane, which results in the lysis of non-nucleated cells such as aged erythrocytes or bacteria. The formation of sublytic pores in nucleated cells can induce cellular activation and/or tissue injury (103). C3b also acts as an opsonin by binding to various surface-expressed receptors, including complement receptor 1 (CR1), CR2, CR3, and CR4 (100).

Although the complement system is a key sentinel of innate immunity, the coagulation system is the main player in hemostasis, both of which are critical to our body's first line of defense against injurious stimuli and foreign invaders. It is, thus, not surprising that there is significant crosstalk between the complement and coagulation cascades (36). The coagulation factors thrombin (67), FXa, FXIa, and plasmin can act as C3 convertases, cleaving both C5 and C3, and robustly generate biologically active C5a and C3a, independent of C3 convertases (6).

The anaphylatoxins C3a and C5a are the main effector molecules of the complement cascades and exert their effector functions through binding and activation of their G-protein-coupled-receptors, C3aR and C5aR1, resulting in the release of pro-inflammatory signals that induce vasodilation, cytokine and chemokine release, neutrophil and macrophage chemoattraction, macrophage activation, T cell and antigen-presenting cell activation, expansion, and survival (100).

Complement activation must be tightly controlled to prevent self-harm. Decay accelerating factor (DAF) or CD55 are membrane-bound regulators that inhibit the early activation of the complement cascade. DAF accelerates the degradation of C3 convertases and competitively prevents the reformation of C3 convertases by interacting with C4b of the classical and lectin pathways and C3b of the alternative pathway, which then interferes with the conversion of C2 to C2b, or factor B to Bb by factor D, thereby preventing the formation of C4b2b C3-convertase and C3bBb C3-convertase, respectively.

By inhibiting the formation of the amplification convertases, DAF effectively blocks downstream amplification and cleavage of the complement cascade (31). Another membrane-bound regulator of complement activation is CD59, which inhibits the assembly of fully functional MAC within the membrane of target cells, by binding to sites on C8 and C9, thus blocking the polymerization of C9 into the complex (29). CR1 is another complement regulatory protein that is variably expressed on the plasma membrane of erythrocytes, eosinophils, monocytes, macrophages, lymphocytes, dendritic cells, Langerhan cells in the skin, and glomerular podocytes (76).

CR1 acts as a cofactor for the Factor I-mediated cleavage and inactivation of C3b and C4b and as an inhibitor of both the classical and alternative pathway convertases (173). Further, human erythrocyte CR1 (E-CR1) is involved in the transport of circulating immune complexes from the circulation to the reticuloendothelial system for clearance (165). Most of the circulating complement proteins are synthesized in the liver.

However, studies have shown that kidney is more than capable of synthesizing most of the activation components of the complement cascade (172). Interestingly, it has been suggested that glomeruli are more vulnerable to insults from circulating complement components, whereas tubular injury is often the result of local complement synthesis, notably via C3 (128).

## Clinical Evidence for Altered Complement in DKD

Multiple complement components have been shown to be altered in the diabetic milieu, with particular complement proteins being associated with a decline in kidney function (summarized in Table 1). Although early evidence indicates that complement proteins may be useful as biomarkers for early DKD and predictors of progression to DKD, complement proteins are not being currently used clinically for diagnosis or to predict prognosis for DKD. The following sections discuss the breadth of complement proteins identified as altered in the systemic circulation or the urine, and differentially expressed in the human kidney.

**Table 1.** Clinical Studies in Which Complement Has Been Explored in Individuals with Diabetic Kidney Disease

Refs.	Biospecimens	Major findings
44, 51, 52, 53, 60, 69	Serum	MBL levels associated with the development and/or progression of albuminuria.
108	Serum	MAp19 concentration associated with increased risk of progression from normoalbuminuria to micro- or macroalbuminuria.
109	Serum	High levels of the complement activator molecule H-ficolin associated with increased incidence of persistent micro- or macroalbuminuria.
150		Acquired loss of E-CR1, DAF, and CD59 in subjects with DKD compared with subjects with non-DKDs undergoing hemodialysis.
41, 120	Plasma	Levels of C3 increased in T2D and associated with increased risk of diabetic complications.
67, 161	Serum	Increased C3 levels were independent indicators of NDRD in T2D.
160	Serum	Decreased serum C3 level was associated with more severe renal insufficiency, higher glomerular histology grading, and poorer renal outcomes, though it failed to be an independent risk factor in T2D patients with DKD.
85	Plasma/urine	Levels of C1q, MBL, Bb, C4d, C3a, C5a, and sC5b-9 were greater in subjects with biopsy-proven DKD; urinary MBL, Bb, C4d, C3a, C5a, and C5b-9 were increased in DKD with urinary C3a, C5a, and C5b-9 positively correlating with urinary protein and eGFR.
133	Plasma	C5b-9, C5a, and C3a were significantly greater in patients with T1D and T2D.
144	Kidney	Increased accumulation of MAC correlated with SMC loss and CML deposition.
134, 152, 155	Kidney	Transcriptomics analysis revealed the complement pathway as one of the most upregulated pathways in DKD, whereas complement regulatory proteins were mostly downregulated.
29, 34, 86, 131, 136, 156, 163	Kidney	Immunohistochemistry revealed increased MBL, MASP1/3, C1q, C3, C3c, C4c, C5a, C3aR, and C5b-9 expression in renal biopsies from subjects with DKD.
17	Kidney	Immunohistochemistry revealed increased C4d and C5b-9 in autopsied kidneys from cases with DKD and correlated with lower eGFR.
127	Kidney	Transcriptomics analysis on postmortem kidneys revealed the complement pathway as the most significantly altered pathway in early DKD, with *C7* being one of the most upregulated genes.
91, 145	Urine	Network and pathway analysis of the urinary proteome in T1D and T2D patients with or without albuminuria identified the complement as one of the top regulated pathways.
61	Urine	Urinary MBL is an independent risk factor with a high predictive power for DKD in T2D patients.
162	Urine	Elevated urinary C3a, C5a, and C5b-9 were independent risk factors for tubular injury in individuals with DKD.
113	Urine	Urinary MAC was present in patients with overt DKD and correlated with eGFR decline.

C3aR, complement C3a receptor; CML, N-ɛ-carboxymethyllysine; DAF, complement decay accelerating factor; DKD, diabetic kidney disease; E-CR1, erythrocytes complement receptor-1; eGFR, estimated glomerular filtration rate; MAC, membrane attack complex; MAp19, mannose binding lectin-associated protein of 19 kDa; MASP1/3, mannose binding lectin-associated serine protease 1/3; MBL, mannose binding lectin; NDRD, nondiabetic renal disease; SMC, smooth muscle cells; T1D, type 1 diabetes; T2D, type 2 diabetes.

### Systemic circulation

High MBL concentrations are associated with increased all-cause mortality in patients with type 1 (115) and 2 diabetes (56). This is of particular interest because the many-fold between-subjects variation in the circulating MBL concentration predominantly is a consequence of polymorphisms in the *MBL2* gene and its promotor and the observed associations, therefore, indicate a causal effect of MBL on mortality in diabetes (137).

The association between MBL and cardiovascular events and mortality in T2D has recently been reassessed in a large cohort study, which indicates a U-shaped association in T2D (48). Earlier studies suggested that MBL levels were significantly greater in T1D patients with DKD when compared with those with normoalbuminuria (57). The presence of high levels of MBL in the early course of T1D was also associated with subsequent development of persistent micro- or macroalbuminuria (64).

Further, in the *post hoc* analysis of a well-characterized Danish case-control cohort of T1D patients, it was observed that high MBL-expressing *MBL2* genotypes were associated with a significantly increased odds ratio of having diabetic nephropathy as compared with patients with low MBL-expressing *MBL2* genotypes (57). A subsequent Finnish observational study of about 1200 patients with T1D added further details to the relationship between MBL and DKD (74).

In line with the observations in the Danish cohort, diabetic patients with macroalbuminuria were found to have higher MBL levels than patients with normoalbuminuria (74). After stratification of the patients into *MBL2* diplotype categories, MBL concentration was found to be higher in two of the six *MBL2* diplotype categories among patients with macroalbuminuria compared with patients with a normal albumin excretion rate. However, in this Finnish cohort, the *MBL2* genotypes or any single SNP were not associated with increased susceptibility to DKD. The discordance between the Finnish study and the Danish study regarding the association between the MBL genotype and DKD may arise by a type 1 error in the Danish study.

However, one must be cautious in directly comparing the two studies, with one study being a case-control study and the other being a cohort study. On the contrary, both studies predominantly included Caucasian patients and used very similar methods in the evaluation of patient characteristics and outcomes. Collectively, these observational clinical studies, therefore, do not show a clear causal relationship between MBL level and development of DKD, but they indicate that MBL could either represent a proxy for a contributing factor or itself be induced by DKD, for example, via inflammation.

Importantly in this regard, it has been shown that the concentrations of both MBL and the inflammatory marker, high-sensitive C-reactive protein (hsCRP), are associated with the progression of renal disease in T1D (55).

The association between MBL and DKD in T2D is less well documented. MBL levels alone or in combination with CRP may predict the progression to albuminuria in T2D patients (56). A more recent study found that in a small cohort of Chinese patients, MBL levels were significantly greater in T2D patients with DKD when compared with those with persistent normoalbuminuria (52).

In a prospective 18-year observational follow-up study of patients with newly diagnosed T1D, increased levels of the complement activating molecule H-ficolin and MBL-associated protein 19 (MAp19) were strongly associated with a greater incidence of progression to microalbuminuria (113, 114). Patients with DKD undergoing hemodialysis were shown to acquire a loss of several important regulators of complement activation, E-CR1, DAF, and CD59, when compared with patients undergoing hemodialysis with nondiabetic renal disease (157).

Reflecting the complexity of the complement pathway, serum C3 has been found to be both increased (45) and decreased in T2D (168). The latter study included a larger cohort of patients with biopsy-proven DKD and showed that the decreased levels of C3 were associated with worse renal function and glomerular injury (168). Further, it is believed that increased serum C3 differentiates nondiabetic renal disease (NDRD) from DKD in patients with T2D (72, 169). Nonetheless, increased concentrations of baseline plasma C3 were associated with an increased risk of DKD in individuals from the general population, as reported in the Copenhagen General Population Study, indicating causality (126).

In another study, plasma C1q, MBL, and Bb were all elevated in patients with DKD when compared with diabetic patients without renal disease (90). Markers of complement activation, such as C4d, C3a, C5a, and C5b-9, were also found to be upregulated in the plasma of patients with DKD (90). Similarly, urinary MBL, Bb, C4d, C3a, C5a, and C5b-9 were all increased in DKD but not C1q. Urinary C3a, C5a, and C5b-9 correlated with urinary protein levels as well as estimated glomerular filtration rate (eGFR) in these patients, suggesting that lectin and alternative pathways are activated in DKD (90).

Overall, the large body of clinical data suggests a contributing role of complement in the pathogenesis of DKD. However, experimental data, including animal studies, are needed to further investigate whether or not the complement system plays a causal role in the development of DKD. In this context, Tan *et al.* (140) recently presented new data, including observations of an increase in complement activation end-products, C5b-9, C5a, and C3a in the plasma of patients with T1D and T2D. Importantly, the increase in these complement activation products was not attenuated by conventional DKD treatment, with the renin angiotensin system (RAS) inhibitors, suggesting that the complement pathway is not therapeutically targeted by currently used therapies for DKD (140).

Moreover, the authors show the pivotal role of complement inhibition at the level of C5a in the prevention of renal damage in an animal model of T1D, as detailed later (preclinical studies) (140). Future clinical studies are, however, needed to test the renoprotective effects of complement modulation in patients with diabetes.

### Kidney

A growing body of evidence indicates differential expression of complement pathway proteins in the kidney in the context of human diabetes. Transcriptome analysis of human kidney biospecimens microdissected into glomerular and tubulointerstital compartments revealed the complement pathway as one of the most significantly upregulated pathways in both the glomerular and tubular compartments in diabetes (163). Notably, *C3* gene expression was significantly upregulated in both glomeruli (163) and tubules (141), with the increase in glomerular *C3* showing a strong correlation with the degree of glomerulosclerosis (163).

In contrast, in a small cohort of patients with T2D, genes associated with the regulation of complement activation such as *DAF* and *CD59* were identified as the most significantly downregulated genes in the glomeruli in diabetes (159). Another study performed transcriptomic analysis on the postmortem human kidney from patients with T2D and early stage DKD, with relatively mild glomerulosclerosis and preserved eGFR (134).

Gene ontology and pathway enrichment analysis identified the complement system as the most significantly activated pathway in early DKD, with *C7* as one of the most upregulated genes (134). This result was further validated by immunohistochemistry, with the expression of C7 elevated in the proximal tubules of the early diabetic kidney, together with an increase in serum C7. Interestingly, other complement products were not increased in the circulation, including C3 and C4, suggesting that C7 might be a biomarker for early DKD (134).

More recently, Wilson *et al.* performed unbiased single-nucleus RNA sequencing of human kidney biospecimens obtained from individuals with diabetes with mild to moderate glomerulosclerosis and interstitial fibrosis, but preserved eGFR, characteristic of early disease (161). This study demonstrated cell-type-specific changes in gene expression crucial for ion transport, angiogenesis, and immune cell activation, with a clear clustering of cells positive for the complement regulatory protein, complement factor H, which was significantly decreased in diabetes (161).

This cluster of cells likely represents parietal epithelial cells. Several studies have used a targeted approach to look at the deposition of complement and complement activation products in the kidney in human DKD. Immunohistochemical staining revealed a significant increase in tubular C5a in renal biopsies from patients with DKD (unspecified types of diabetes), but no changes in C5aR1 (164). In contrast, immunostaining for C3aR was found to be upregulated in renal biopsies from patients with DKD when compared with nondiabetic controls and was co-localized with CD68-positive macrophages in the tubulointerstitium (91). The upregulation of C3aR was correlated with the severity of glomerulosclerosis and interstitial damage (91).

Deposition of the complement activation product, C5b-9 was increased in human DKD with both T1D (38) and T2D (171). In human T2D, the increased immunolabeling of C5b-9 in the tubular and interstitial area was correlated with an increase in MBL and mannan-binding lectin serine protease 1/3s (MASP1/3) expression in the tubulointerstitial area as well as the degree of tubulointerstitial damage, suggesting a role for MBL pathway-induced local activation of the complement system in DKD (171).

Further, evidence of activation of the classical pathway was also presented in DKD with an increase in the deposition of glomerular C1q, which was accompanied by worse renal outcomes [increased proteinuria and reduced eGFR in these patients (138, 143)]. More recently, it was found that kidney biopsies from T2D patients who exhibited C4c deposits with a concomitant increase in C1q deposition were associated with increased proteinuria and interstitial inflammation score, as well as poorer survival rates (33).

The accumulation of MAC has been associated with the loss of medial smooth muscle cells in intrarenal muscular arteries of patients with T2D, likely due to an increase in the glycoxidation of the MAC inhibitor, CD59, resulting in an increased lytic action of MAC, and this was associated with more severe glomerulosclerosis (151).

Finally, in a study looking at autopsied kidneys from patients with T1D or T2D, immunostaining for complement activation products, C4d and C5b-9 was significantly increased in the DKD cohort and this increase was even more prominent in T1D than in T2D kidneys (20). However, whether the difference in the degree of complement activation in T1D or T2D was due to different activation pathways or the duration of diabetes was unclear.

### Urine

Network and pathway analysis of the urinary proteome in T1D and T2D patients with or without albuminuria has again identified the complement as one of the top regulated pathways in diabetes (96, 152). Urinary MBL levels are found to be independent risk factors, with a high predictive power for DKD in T2D patients (65). Interestingly, MAC was detected in the urine from patients with microalbuminuria and macroalbuminuria but not in the control group.

Other complement factors such as C5, C5a, C5b, factor H, and C8 were only detected in patients with macroalbuminuria, further suggesting a role for complement in the progression of DKD (96). Increased concentrations of urinary CD59 were associated with a lower risk of ESRD and all-cause mortality, whereas increased concentrations of factor H-related gene 2 (FHR2) were associated with a higher risk of all-cause mortality in people with proteinuric DKD (152). The increase in urinary C3a, C5a, and C5b-9 correlated with a more advanced-stage DKD in T2D, including more severe proteinuria, renal tubular damage, and infiltration of interstitial infiltration cells (170). Further, it was reported that urinary MAC was present in overt DKD at levels comparable to autoimmune glomerulonephritis and it correlated with GFR decline (118).

## Preclinical Data

Similar to human studies, complement and coagulation cascades are identified as being within the top regulated pathways in the kidney in models of diabetes. In glomeruli of BTBR ob/ob diabetic mice at 24 weeks of age, *C3* was identified as one of the top upregulated differentially expressed genes (DEGs), and *Daf* and *Cd59* were identified as the top downregulated DEGs in the diabetic glomeruli (25).

Transcriptomics performed in the kidneys of Zucker fatty diabetic/spontaneous hypertensive heart failure (ZS) rats also revealed *C3* as one of the most upregulated genes when compared with nondiabetic lean counterparts, with other components of the complement cascade also upregulated, including *C1q*, *C2*, *C3*, *C4*, *C6*, *C8*, and *C9* (75). Although *C5* and *C5ar1* were not detected by unbiased transcriptomics (RNASeq), targeted detection using quantitative reverse transcription PCR revealed an increased expression of *C5* and *C5ar1* in the kidney of the diabetic ZS rats when compared with nondiabetic lean controls (75).

In another rat model of T2D (high-fat diet combined with low-dose streptozotocin) C1q, MBL, MASP-2, B factor, C3, and C5b-9 were found to be mainly expressed in the tubules and their expression was significantly increased in diabetic rats with increased albuminuria and renal injury (66).

Induction of diabetes with streptozotocin in female wildtype (WT) C57BL/6J and *C3*^−/−^ mice showed that the lack of *C3* attenuated the diabetes-induced increase of kidney-to-body weight ratio and glomerular basement membrane thickness in the diabetic kidneys (112). Further, diabetes led to an increase in renal collagen IV mRNA expression in the WT animals, whereas no difference was observed between *C3-*deficient diabetic animals and their *C3-*deficient nondiabetic controls (112). More recently, the role of C3aR in DKD was examined by using *C3ar*^−/−^ mice in a model of T2D (high-fat diet and streptozotocin) (91).

It was found that albuminuria and glomerulosclerosis were significantly attenuated in mice lacking *C3ar* when compared with WT diabetic C57BL/6J mice (91). Further, a role for C5aR1 in DKD has also been examined in a streptozotocin-induced mouse model of T1D (140). It was found that genetic deletion of *C5ar1* reduced albuminuria by 75% in diabetic mice when compared with WT.

In addition, in the absence of *C5ar1*, oxidative stress and renal inflammation were attenuated in diabetes as reflected by reduced urinary 8-isoprostanes, and reduced infiltration of tubulointerstitial macrophages, as well as a restoration of the anti-inflammatory regulatory T cells (Tregs) (140).

Other components of the complement system have also been investigated in experimental models of diabetes. Circulating and glomerular MBL was significantly elevated in streptozotocin-induced diabetic mice (108, 111). MBL deficiency attenuates renal hypertrophy and glomerular enlargement in mice with streptozotocin-induced diabetes (107, 109). Diabetes-induced albuminuria was attenuated by MBL deficiency in the first of these studies using knockout mice backcrossed for six generations onto a C57BL/6J background (107).

However, it was also found that the urinary albumin excretion was higher in nondiabetic *MBL-*deficient animals as compared with nondiabetic WT mice. In a later study using *MBL*-deficient mice backcrossed for >12 generations onto a C57BL/6J background as well as onto a 129S6 background, diabetes-induced albuminuria was not affected by MBL in any of these mouse stains (109). Also, in this later study, albumin excretion was not affected by MBL *per se* in the two-way ANOVA measuring the separate effect of knockout status (109).

Further, diabetes was associated with an increase in the autoreactivity activity of MBL in the kidney, further suggesting an activation of the lectin pathway in the diabetic kidney (13, 14, 111). More recently, in streptozotocin-induced diabetic BALB/c mice, albuminuria was worse in *Daf* knockout mice when compared with WT controls (9). Further, similar findings were observed with targeted deletion of *Daf* in the podocyte using the podocin-cre mouse model (9).

Taken together, these clinical and preclinical studies provide unequivocal evidence for complement overactivation and dysregulation in DKD and this is observed irrespective of the experimental approach; whether it be via targeted profiling of complement proteins or by using unbiased global discovery where a complement signal is identified, the latter, more powerful approach providing remarkable confidence that complement as a network may play a key role in DKD development and progression.

## Mechanisms by Which Complement Is Activated in Diabetes

**FIG. 2.**
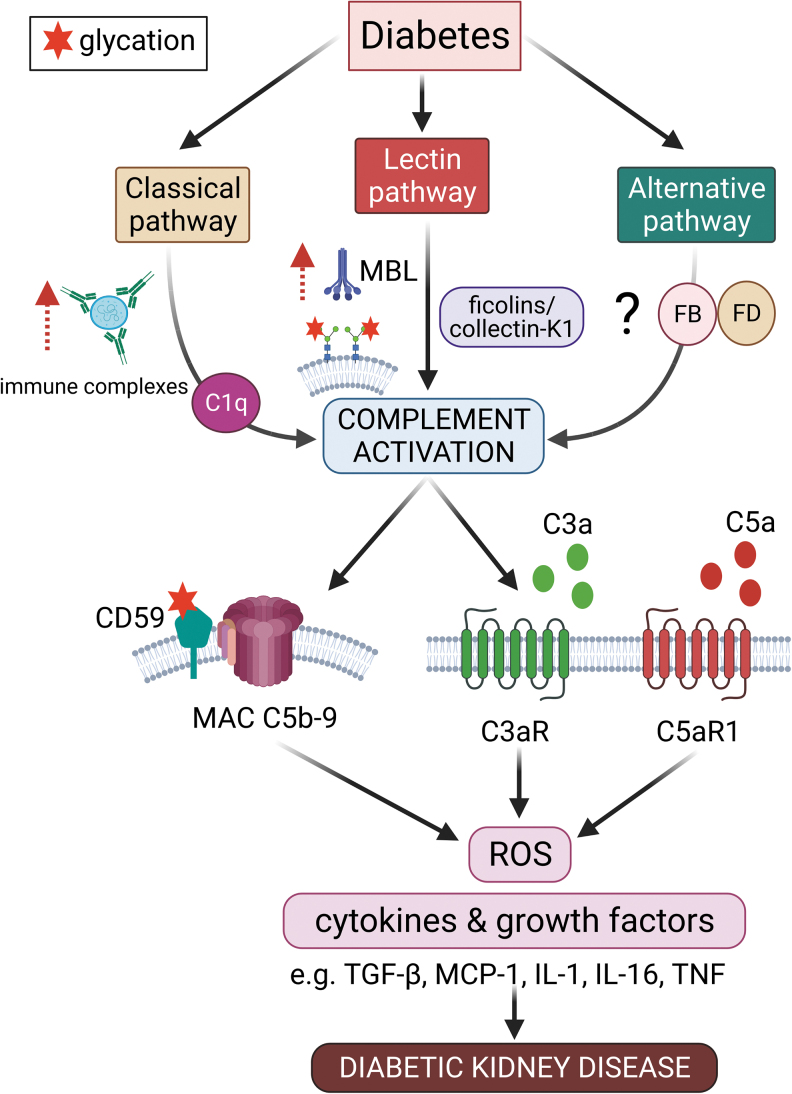
**Activation of the complement cascade in diabetes, leading to diabetic kidney disease.** In diabetes, the classical and the MBL pathways have been shown to be activated with the increased deposition of immune complexes in the kidneys and increased autoreactivity to MBL due to accelerated protein glycation, respectively. However, evidence for the activation of the alternative pathway is less clear in diabetic kidney disease. Increased glycation of regulatory protein, CD59, leads to a reduced inhibitory action on MAC in diabetes, exacerbating complement auto-attack. Increase in complement activation leads to the production of anaphylatoxins, C3a and C5a, which act on their respective receptors, C3aR and C5aR1, and result in the production of ROS, inflammatory cytokines, and growth factors, such as TGF-β, MCP-1, IL-1, IL-16, and TNF, which are mediators of renal fibrosis and inflammation in diabetic kidney disease. IL, interleukin; MCP-1, monocyte chemoattractant protein-1; ROS, reactive oxygen species; TGF-β, transforming growth factor-β; TNF, tumor necrosis factor. Color images are available online.

This brings us to the next section of our discussion, which will focus on how particular complement pathways are activated in DKD. Currently, there are two key proposed mechanisms describing how the complement system is activated in DKD: (1) binding of lectin pathway pattern recognition molecules to altered proteins due to accelerated protein glycation in diabetes (44, 58); and (2) increased glycation of complement regulatory proteins as a result of high glucose, leading to reduced regulatory capacity stimulating complement auto-attack (1, 124) (Fig. 2).

Østergaard *et al.* have reported an increase in MBL autoreactivity toward kidney tissue in diabetes (13, 14, 111). MBL is a recognition molecule that is able to distinguish between carbohydrates found on self-glycoproteins and the carbohydrate patterns found on infectious nonself surfaces known as pathogen-associated molecular patterns (PAMPs), or the altered self as a result of damage to self-cells known as damage or danger-associated molecular patterns (DAMPs) (47).

Normal human glycoproteins are not recognized with a sufficiently high binding strength by MBL. In diabetes, glycation occurs when glucose and other sugars form covalent adducts with plasma proteins through a nonenzymatic process leading to the formation of Amadori products, which eventually form AGEs, which contribute to the development of diabetic complications (42). The formation of these high-density glycated proteins on cell membranes induces MBL binding, which then activates the lectin pathway (44). Indeed, it has been found that products of complement activation were co-localized with glycated proteins in human diabetic kidneys (151).

Complement activation is tightly regulated by plasma and membrane regulatory proteins (49). CD59 is a specific inhibitor of MAC formation that restricts C9 polymerization. Site-directed mutagenesis studies on human CD59 revealed that the active site of CD59 contains a glycation motif formed by amino acid residues K41 and H44 and that glycation at these sites could render CD59 inactive (1). In human endothelial cells, the glycation of CD59 inhibits its MAC inhibitory function, subsequently increasing the susceptibility to MAC-induced release of growth factors (1).

Further, it was found that a significant fraction of CD59 present in the urine of a small cohort of diabetic subjects was glycated, and this was not the case in the urine of nondiabetics, further suggesting that the inhibitory action of CD59 was compromised, resulting in hyperactivation of the complement system (1). In addition, later studies from the same group showed that glycated CD59 was found to be co-localized with MAC in human diabetic kidneys with a reduction in the activity of CD59 in erythrocytes from diabetic subjects, a finding consistent with glycation inactivation of CD59 *in vivo* (124).

## Known and Putative Mechanisms by Which Complement Is Involved in the Pathogenesis of DKD

### Membrane attack complex

MAC and its lytic pore are undoubtedly the most recognized effectors of the complement cascades. On nucleated cells, MAC is tightly regulated by regulatory proteins such as CD59, assembled MAC complexes are actively removed from the membrane, and the lytic action of MAC is counteracted by ion pumps (103). However, the sublytic action of MAC is not without consequences. The assembly of pores at sublytic levels in the nucleated cell membrane can trigger a series of pro-inflammatory responses, including the release of inflammatory cytokines inducing cellular activation and tissue injury (103).

Further, the insertion of sublytic MAC pores into cell membranes can trigger the release of growth factors and cytokines such as basic fibroblast growth factors, interleukin-1 (IL-1), monocyte chemoattractant protein-1 (MCP-1), and platelet-derived growth factor (PDGF) that promote cell proliferation, inflammation, and thrombosis (49). In glomerular mesangial cells, the insertion of sublytic MAC results in increased production of ROS (2); collagen IV and fibronectin (148, 156); prostaglandin E, IL-1, and tumor necrosis factor (TNF) (131); and the release of IL-16, TGF-β and differentiation of CD4^+^ T cells into Th17 cells (167).

### Anaphylatoxin C3a and C5a

The anaphylatoxin receptors are widely expressed throughout the kidney. C3aR is expressed in glomerular epithelial cells and proximal tubular cells in the normal human kidney, but not in mesangial or glomerular endothelial cells (18, 39). C5aR1 is expressed in mesangial cells, podocytes, and proximal tubular epithelial cells (30, 39, 83). Both C3aR and C5aR are expressed on cells of myeloid lineage, such as macrophages, monocytes, neutrophils, basophils, and eosinophils in mice and humans (83). The podocyte is found to express the majority of the complement components, including C3, C3aR, and C5aR, as well as components that are important for the formation of MAC such as C4, C5, C6, C8, and C9 (89).

C3a and C5a can induce tubular epithelial–myofibroblast transdifferentiation via upregulation of their respective receptors, C3aR and C5aR1, and the TGF-β/CTGF pathways in human proximal tubular cells (94). *In vitro* experiments have shown that C3a stimulated the production of collagen I (18) and TGF-β in proximal tubular cells (117). Similarly, murine cortical tubule cells stimulated with C5a secreted TGF-β and this response was blocked by the addition of a C5aR1 antagonist, JPE-1375 (17).

In mouse primary tubular cells stimulated with C5a, vimentin, KIM-1, and IL-10 expression was increased, as well as secretion of MCP-1 and TGF-β and these responses were abrogated in the absence of C5aR1 or C5L2 (97). In addition, C5a is an epigenetic mediator, triggering the hypomethylation of genes that are involved, at least in part, in the Wnt/β-catenin signaling, such as *BCL9*, *CYP1B1*, and *CDK6*, in human renal tubular epithelial cells (23). However, whether these processes occur in renal cells under the conditions of high glucose and contribute to renal injury in diabetes warrants further investigation.

### The inflammasome

The activation of the complement in diabetes may be linked with yet another component of the body's first line defense called inflammasomes, through the formation of nonlytic membrane-attack complexes on self-cells as well as mediated by the anaphylatoxins C3a and C5a generated by complement activation. The nucleotide-binding oligomerization domain (NOD)-like receptors (NLRs) are another type of pattern-recognition receptors that are activated in the innate immune system's response to DAMPs and PAMPs and induce inflammasome activation.

The NOD-, leucine-rich repeats- and pyrin domain-containing 3 (NLRP3) inflammasome is a cytosolic multiprotein complex that is activated by a priming signal (Signal 1) followed by an activating signal (Signal 2), but the precise mechanism remains incompletely understood. Inflammasome activation is a central element in the pathophysiology of a range of chronic diseases, including cryoporin-associated periodic syndromes, gout, inflammatory bowel diseases, liver fibrosis, and possibly also complications of diabetes (99, 154).

It is established that the assembly of the canonical, multimolecular NLRP3 inflammasome consisting of NLRP3, apoptosis-associated speck-like protein containing a caspase recruitment domain (ASC) and pro-caspase 1 causes activation of the NLRP3 inflammasome generating active caspase 1. Once activated from pro-caspase 1, caspase 1, in turn, cleaves pro-IL-1β and pro-IL-18 to mature IL-1β and IL-18, both of which have been linked to renal injury (68).

There is likewise increasing evidence that supports a central role of the NLRP3 inflammasome in DKD (79, 84, 105) whereas other inflammasomes (NLRP1, NLRC4, AIM2) have not been explored in detail.

The mechanistic basis of a link between complement and inflammasome activation has been investigated in several studies, as previously reviewed (10). In a study using lung epithelial cells, the effects of the transmembrane pore-forming MAC on NLRP3 inflammasome activation were studied (149). The cells were treated with neutralizing antibodies toward CD59, which is a membrane-bound endogenous negative complement regulator inhibiting C9 polymerization to C5b-8, to allow MAC assembly on the cell surfaces (149).

It was observed that exposure to sublytic concentrations of normal human serum containing the complement led to inflammasome activation, including caspase 1 activation, and the release of IL-1β. However, no inflammasome activation was seen when using C5, C7, or C9 depleted serum, which, therefore, indicates that the formation of the MAC is necessary for the observed inflammasome activation by the complement (149).

This complement-induced inflammasome activation was further shown to be mediated via an increase in the intracellular Ca^2+^ concentration, which is a known trigger of NLRP3 inflammasome activation (149). Additional experiments with the siRNA-mediated knockdown of *Nlpr3* or *Asc* showed that silencing these components of the canonical NLRP3 inflammasome blocked the complement-mediated IL-1β release (149).

In another study, bone-marrow derived dendritic cells were primed with lipopolysaccharide (LPS) to stimulate the expression of pro-IL-1β as Signal 1 of NLRP3 inflammasome activation and subsequently exposed to the complement. In these experiments, exposure to the complement led to caspase 1 activation, which occurred on the assembly of the NLRP3 inflammasome as well as in the secretion of mature IL-1β, which is produced from pro-IL-1β by cleavage by activated caspase 1 (81). Of note, these effects were observed by using complement concentrations that did not increase cell death.

In additional experiments comparing WT dendritic cells with *Nlpr3*^−/−^ and *Asc*^−/−^ dendritic cells as well as pharmacological caspase 1 inhibition, it was confirmed that the NLPR3 is required for complement-mediated IL-1β and IL-18 maturation, which was dependent on potassium efflux and calcium influx (81). These results were further substantiated by *in vivo* studies showing complement-mediated IL-1β and IL-18 secretion by NLRP3-dependent and ASC-dependent mechanisms (81).

Additional *in vivo* experiments comparing complement-mediated inflammasome activation in mice deficient of the C3a receptor or the C5a receptor with WT animals found only a minor attenuation of the complement-mediated inflammasome activation by deficiency of these receptors of the complement anaphylatoxins C3a and C5a, when the cells were stimulated with LPS as based on the effects on serum IL-1β.

However, the inhibition of MAC formation by using C6-neutralizing antibodies or C6-deficient mice was seen to suppress serum IL-1β levels, which, therefore, indicates that the complement-induced activation of the NLRP3 inflammasome is driven via MAC formation and not through the C3a and/or C5a receptors.

However, other studies suggest that C3a signaling through the C3a receptor may, in fact, also induce NLRP3 inflammasome activation. Already in 1987, Haeffner-Cavaillon *et al.* reported that stimulation of human monocytes with the C3a metabolite C3adesArg resulted in IL-1β production and release, which, therefore, indicates the activation of the NLRP3 inflammasome as it controls IL-1β maturation (53). Further, it was also shown that C3a signaling through the C3a receptor can act as Signal 2 for the NLPR3 inflammasome in LPS primed (Signal 1) human monocytes, macrophages, and dendritic cells by mechanisms mediated by a cellular ERK1/2-dependent adenosine triphosphate (ATP) efflux (12).

The C5-derived anaphylatoxin C5a has also been studied as a putative regulator of NLRP3 inflammasome activation. It was shown in *in vitro* studies that C5a augments the activation of the NLPR3 inflammasome by acting synergistically with TNF as a Signal 1 to induce the expression of pro-IL-1β in human peripheral blood mononuclear cells (129). Further, *in vitro* studies of murine bone marrow cells have showed that C5a augments the LPS-induced production of pro-IL-1β whereas a paradoxical decrease in pro-IL-1β expression was seen in macrophages (54).

In addition, in a similar model using macrophages and bone marrow cells from *C5ar1*^−/−^ mice, it was observed that the impact of C5a was mediated via C5aR1 in bone marrow cells, but not in macrophages (54). In additional *in vivo* studies, the authors found that the LPS-induced increase in plasma IL-1β concentration was attenuated in *C5ar1*^−/−^ mice compared with WT mice, which highlights the importance of C5a as an activator of the NLRP3 inflammasome during endotoxemia via the C5aR1 at a systemic level (54).

As previously reviewed, the effects of C5a are most likely, at least in part, mediated via the induction of nuclear factor kappa B (NF-κB) activation as well as C5a receptor 1 activation and ROS production (10). In a later study, the impact of the C5a receptor 2, C5aR2, was investigated by comparing IL-1β production in LPS-primed peritoneal macrophages from WT mice and *C5ar2*^−/−^ mice (166). From these experiments, it was seen that C5aR2 deficiency resulted in a marked attenuation of IL-1β production after NLRP3 inflammasome activation by LPS priming and activation using ATP, nigericin, or monosodium urate as Signal 2 (166).

This article does not describe in which media these *in vitro* experiments were performed, including if the complement containing serum with complement factor C5 was present, which therefore limits the possibility to deduce the role of C5a, although C5a was shown to activate protein kinase R (PKR), a suggested mediator of NLRP3 inflammasome activation. However, the role of C5aR2 for inflammasome activation was supported by measuring peritoneal inflammation *in vivo* (166).

**FIG. 3.**
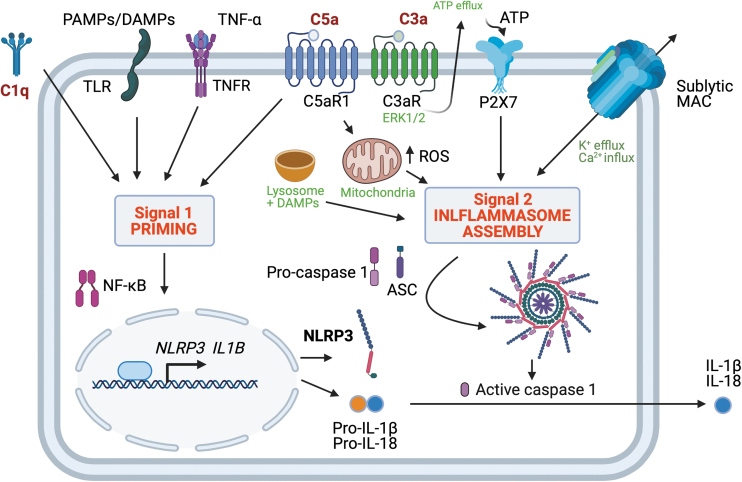
**NLRP3 inflammasome signaling via complement.** Activation of the NLRP3 inflammasome requires a priming signal and an assembly signal. Signal 1: The priming signal is induced by PAMP/DAMP recognition by pattern recognition receptors (*e.g.*, TLRs) and pro-inflammatory cytokines (TNF-α) and promotes nuclear translocation of NF-κB, with subsequent NLRP3 and IL-1B gene transcription. Signal 2 promotes the assembly of NLRP3, ASC, and caspase-1 complexes to form an active NLRP3 inflammasome, where active caspase-1 processes proIL-1β/proIL-18 into mature IL-1β/IL-18. Complement components C1q and C5aR1 potentiate Signal 1. C5aR1 act as a priming signal to sustain inflammasome activation during the uptake of DAMPs, using a mechanism that involves lysosomal damage and release of cathepsin B. Direct activation of C5aR1 promotes Signal 2 for NLRP3 inflammasome activation via changes to mitochondrial metabolism and ROS production. C3aR is believed to regulate ATP efflux via P2X7 engagement, and sublytic MAC formation enhances intracellular Ca^2+^. Figure adapted from Arbore and Kemper (10). ASC, apoptosis-associated speck-like protein containing a caspase recruitment domain; ATP, adenosine triphosphate; DAMPs, danger-associated molecular patterns; NF-κB, nuclear factor kappa B; NLRP3, NOD-, leucine-rich repeats- and pyrin domain-containing 3; PAMP, pathogen-associated molecular pattern; TLRs, toll like receptor. Color images are available online.

The mechanisms of complement-induced inflammasome activation (Fig. 3) may, therefore, prove to differ between cell types and species, including either signaling by the complement anaphylatoxins or sublytic MAC deposition on self-cells (12).

### Mitochondrial homeostasis and immunometabolism

The crosstalk between metabolism and immunity, or immunometabolism, is an exciting new field of research that investigates how metabolic reprogramming in diseases has an impact on immune cell activation, proliferation, mobilization, and acquisition of effector or regulatory functions (95). The interconnection between immunometabolism and diabetes has recently been reviewed elsewhere (16, 63). The involvement of the complement system in basic cellular processes, particularly those of a metabolic nature, has been previously described (10, 61, 78).

It is, thus, not surprising that the complement system may also be involved in modulating immunometabolism in DKD. Microarray analysis on glomerular mRNA from BTBR *ob/ob* mice revealed that the top dysregulated KEGG pathways in diabetes are involved in cellular metabolism, including metabolic pathways, biosynthesis of unsaturated fatty acid, the peroxisome proliferator-activated receptor (PPAR) signaling pathway, and fatty acid metabolism, as well as the complement and coagulation cascades, which further suggests an association between complement activation and cellular metabolism (25).

Mitochondria have been shown to play a central role in regulating immunometabolism via several mechanisms (8): First, alterations in metabolic pathways (tricarboxylic acid [TCA] cycle, oxidative phosphorylation, and fatty acid β-oxidation) induce transcriptional changes that influence the activation, differentiation, and survival of immune cells. Second, mitochondria can activate inflammatory responses via the inflammasome. Third, fission and fusion of mitochondria can influence immune function; and fourth, localization of mitochondria in close proximity to the endoplasmic reticulum in immune cells can affect immune cell metabolism (8). Indeed, previous studies have demonstrated a causal link between activation of the complement system and modulation of mitochondrial homeostasis in different cellular systems (98, 144).

A recent review from the Kemper group has summarized current knowledge about the intracellularly active complement system and its relationship with mitochondria (125).

Our group has previously shown that stimulation of human proximal tubule cells with C5a induced changes in mitochondrial respiration after trifluoromethoxy carbonylcyanide phenylhydrazone (FCCP)-stimulated uncoupling and ATP-linked respiration (140). Further, transcriptomics of kidney cortex from streptozotocin-induced diabetic mice treated with the specific C5aR1 antagonist, PMX53, revealed that 5 out of the top 10 regulated Reactome pathways were involved in cellular metabolism, including glycolysis, mitochondrial fatty acid β-oxidation, and fatty acyl-CoA biosynthesis (140).

**FIG. 4.**
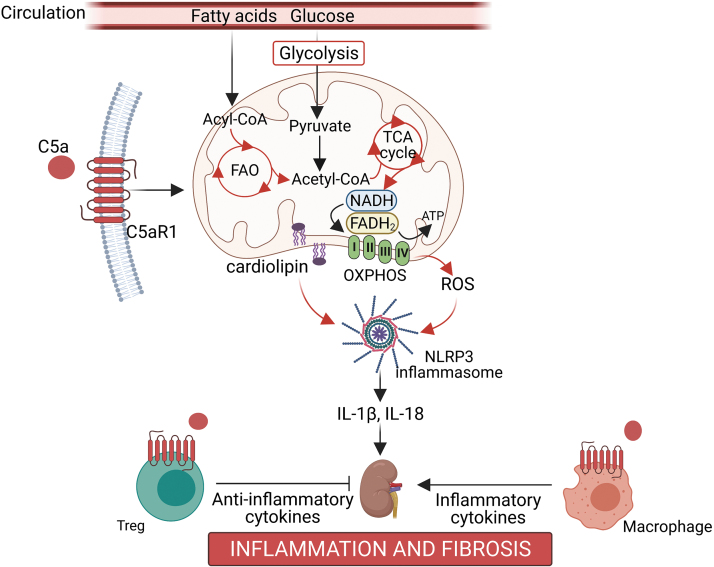
**Complement C5a-mediated immunometabolism.** Intrarenal activation of complement C5a receptor 1 (C5aR1) leads to a change in genes associated with cellular metabolism such as glycolysis, FAO, and TCA cycle (circled in *red*). These changes lead to changes in mitochondrial metabolism and mitochondrial lipid composition such as cardiolipin remodeling. Changes to cardiolipin composition of the mitochondrial membrane can lead to the activation of the NLRP3 inflammasome, thus activating the inflammatory response via IL-1β and IL-18. In addition, activation of C5aR1 on Tregs inhibits its anti-inflammatory function and lowers FoxP3 expression. On the other hand, activation of C5aR1 on macrophages may induce its polarization to the inflammatory M1 form, inducing inflammation. Both these exacerbate inflammatory responses in the kidney, leading to renal inflammation and fibrosis. FAO, fatty acid β-oxidation; TCA, tricarboxylic acid; Tregs, regulatory T cells. Color images are available online.

Interrogation of the lipidomics signature revealed abnormal cardiolipin remodeling in the diabetic kidney, a cardinal sign of disrupted mitochondrial architecture and bioenergetics, and these changes were restored by PMX53 treatment (140). Of note, a previous study has found that mitochondrial cardiolipin is required for NLRP3 inflammasome activation (69). Our study also found that the inhibition of C5aR1 with PMX53 not only reduced albuminuria and glomerulosclerosis, but it also led to the resolution of inflammation as shown by decreased infiltration of macrophages into the tubulointerstitium and restoration of the loss of Tregs in the diabetic kidney (140). This mechanism is illustrated in Figure 4.

Morigi *et al.* recently reported a role for C3aR in disrupting podocyte mitochondrial homeostasis, contributing to DKD (104). Using the BTBR *ob/ob* leptin-deficient mouse model, an upregulation of glomerular C3 and C3aR was observed along with features consistent with a change in mitochondrial morphology, including swelling of the mitochondrial matrix in podocytes (though representative electron microscopy images only were shown) and a decrease in glomerular immunostaining of mitochondrial-associated proteins including VDAC, ATP51 (a subunit of the ATP synthase) and SOD2 expression, suggesting a disruption in mitochondrial function (104).

The changes cited earlier were reversed by treatment with a C3aR antagonist, SB290157, along with improvement in albuminuria. Closer investigation in cultured human podocytes exposed to C3a revealed mitochondrial fragmentation, a reduction in inner mitochondrial membrane potential, decreased ATP content, and a decrease in citrate synthase activity, which is the initial enzyme in the TCA cycle (104). Collectively, these studies highlight a role for C5aR1 and C3aR in mediating immunometabolism in the pathogenesis of DKD.

### Gut–kidney axis

One increasingly recognized putative mechanism that may contribute to kidney injury in diabetes is the microbiota, via the gut–kidney axis. The gut microbiota is altered during diabetes with a reduction in species that produce the beneficial short chain fatty acid butyrate (73, 88, 123, 153). Butyrate enhances the integrity of the gastrointestinal epithelial barrier (40, 120, 158), via an increase in GLP-2 secretion by intestinal L cells (37) and activation of local Tregs (133).

The state of diabetes is associated with increased intestinal permeability, permitting the translocation of bacterial endotoxin, also referred to as LPS, into the systemic circulation (4, 27, 59, 122). This metabolic endotoxemia contributes to the chronic low-grade inflammation that is present in the diabetic milieu, and it is now believed to be a contributor to DKD (106). Recently, there has been great interest in therapeutic options to target the gut–kidney axis, including by dietary modifications (135), and this could serve to limit the increased permeability observed with diabetes and to limit the downstream kidney injury in DKD.

Although complement components in the systemic circulation are predominantly synthesized by the liver, intestinal epithelial cells are also capable of synthesizing the complement. Both C3 and C4 transcripts have been observed in the small intestinal crypts in biopsies from both healthy patients and patients with Crohn's disease (82), and C3 was present in the cultured intestinal tissue from healthy individuals (80).

Similarly, a recent study observed upregulation of C3, but not C5 expression in both inflamed and noninflamed (remission) biopsies from patients with Crohn's disease compared with healthy controls (139). In Caco-2 cells, C3, C4, and factor B expression has been demonstrated, with C3 expression increasing dose-dependently by either IL-1β or TNF-α (7). In mice, C3, but not C4 expression was observed, with C3 expression higher in the colon compared with the ileum (139). C3 deposition has been observed on commensal bacteria from the gastrointestinal lumen (77, 139), and C3b has been observed coating the apical membrane of the distal colon in a model of induced colitis (93), indicating that the complement is activated in the gastrointestinal tract.

The intestinal epithelial cell lines Caco-2, HT-29, and T48 express the CRs C3aR on the apical membrane (102), indicating that intestinal epithelial cells may respond to the luminally activated complement C3a. Toll like receptor 4 (TLR4) activation induces C3 expression by intestinal epithelial cells (121, 139), providing a feedback loop whereby recognition of bacteria by the enterocytes increases complement production and subsequent activation in the intestinal lumen. The intestines are a source of complement, particularly C3, which is secreted into the lumen and activated in response to bacterial invasion of the inner mucosal barrier.

As described earlier, increased intestinal permeability is observed in diabetes, and diabetes is associated with activation of the complement system. It has been demonstrated that both C3a and C5a increase monolayer permeability of intestinal epithelial cells *in vitro*, via activation of the ERK pathway and production of IL-8 (21, 102). It is well established in animal models of inflammatory bowel disease that blockade of the complement components C3a and C5a limits intestinal inflammation. Genetic deletion of *C3ar* improves clinical scores in dextran sulfate sodium (DSS) colitis (160), and treatment with C5aR antagonists (70) or C5a neutralizing antibodies (24) reduces induced colitis in animal models.

Less research has been conducted outside the realm of intestinal inflammatory diseases. It was recently demonstrated that diabetic (*db*/*db*) mice have increased serum, renal, and intestinal C5a protein compared with their nondiabetic counterparts (86). This study also showed a decrease in the tight junction protein claudin-1 in the colon with diabetes, which was partially restored with a C5aR antagonist (86).

**FIG. 5.**
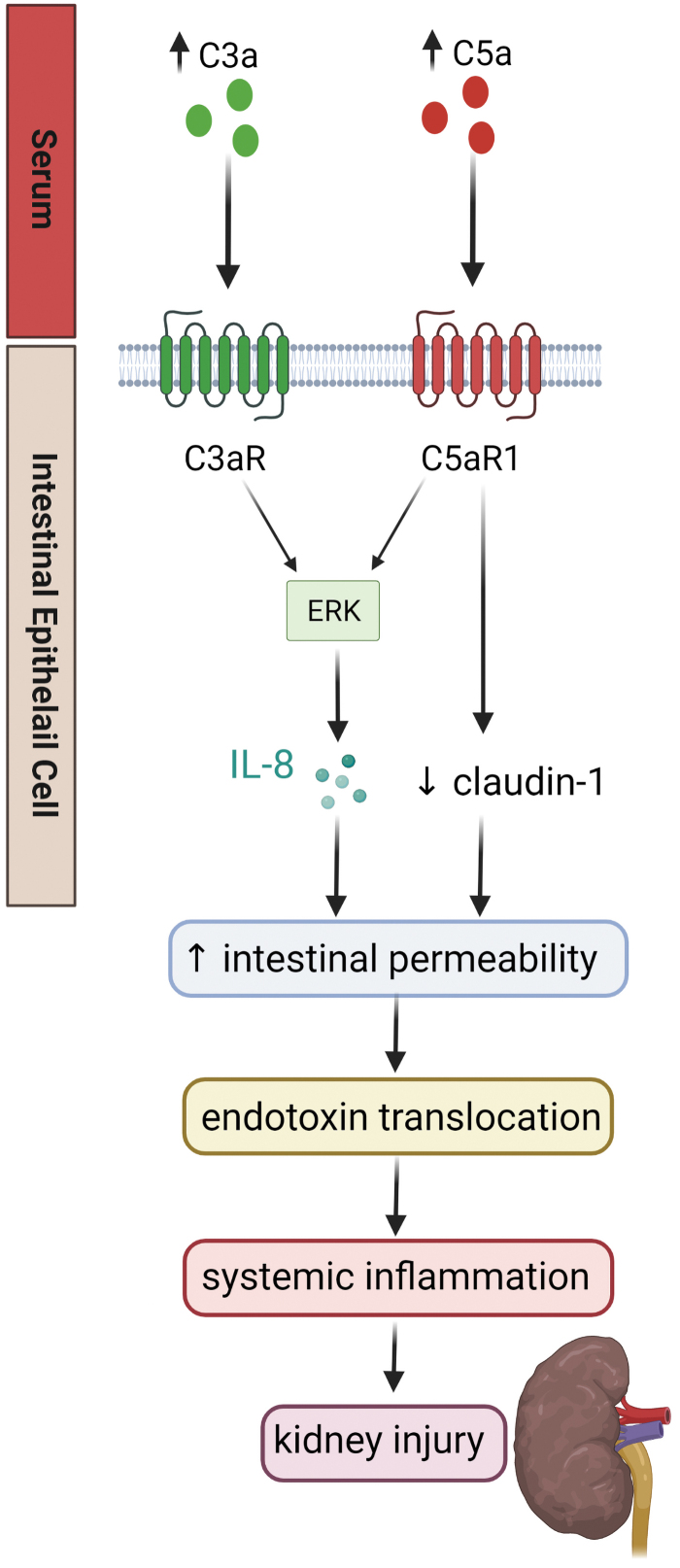
**Complement and the gut**–**kidney axis.** During diabetes, there is an increase in serum levels of the anaphylatoxins, C3a and C5a, which ligate C3aR and C5aR1, respectively, on intestinal epithelial cells, leading to activation of the ERK pathway and IL-8 production. It is believed that this may be able to promote increased intestinal permeability. There is evidence that C5aR1 ligation decreases claudin-1 expression. The increased intestinal permeability permits the translocation of bacterial endotoxin and systemic inflammation, which may contribute to renal dysfunction. Whether C3a and C5a have direct effects at the level of the gut, or further downstream in diabetes has not yet been fully elucidated. Color images are available online.

Although it does not appear that the intestines are a source of C5a, the cell lines Caco, HT-29, and T48 express C5aR1 and C5L2 (21), suggesting that the intestines may respond to C5a from the systemic circulation. Further, overconsumption of highly processed diets can activate the complement pathway and contribute to DKD via the gut–kidney axis (136) (Fig. 5).

### Complement and oxidative stress

Another less well-explored mechanism of complement-mediated renal injury in diabetes is oxidative stress, which is somewhat surprising considering that the complement is associated with mitochondrial dysfunction and the mitochondrial respiratory chain is a key site at which ROS can be generated. Although previous preclinical studies have shown an association between complement pathway activation and increased production of ROS (2, 140, 150), there is a lack of mechanistic understanding of how complement end products such as MAC and the anaphylatoxins, C3a and C5a, produce ROS and the types of ROS involved, especially in the context of diabetes and DKD.

Within mammalian cells, there are several sources of ROS, including those generated from within the mitochondria and from within the cytosol, including the enzyme system nicotinamide adenine dinucleotide phosphate oxidase (NOX) (26). In particular, mitochondria are believed to be the main site of intracellular ROS generation via respiratory complexes, contributing ∼80% of superoxide in the basal state (26). As previously alluded, complement is intrinsic to immunometabolism largely via its indirect effects on mitochondria (125).

Thus, it would not be surprising that the complement may affect mitochondrial function and ROS production. Indeed, intracellular C5aR1 activation was shown to induce NLRP3 inflammasome activation in CD4^+^ T cells via the generation of mitochondrial ROS, which act as signals 1 and 2 for the inflammasome (11). Nonetheless, much is still unknown in terms of the precise mechanisms of how the complement may regulate ROS production within the mitochondria, especially in nonimmune cells such as renal cells.

On the other hand, the family of NADPH oxidases (Nox enzymes) is another important source of ROS. One of the earliest studies to investigate complement-stimulated ROS production was performed by Adler *et al.*, who stimulated rat glomerular mesangial cells with MAC (formed from the purified human complement components C5b6 and normal human serum) and measured ROS production (2). It was shown that MAC stimulated the formation of both superoxide and hydrogen peroxide in glomerular mesangial cells. Interestingly, the soluble form of decayed MAC did not significantly increase ROS formation, suggesting that membrane interaction of the MAC is a requirement for stimulating the cellular production of ROS (2).

In another study, superoxide was generated from rat glomerular epithelial cells stimulated with sheep anti-Fx1A antiserum and normal horse serum to form MAC (119). The MAC-stimulated superoxide production was effectively blocked by an NOX inhibitor, diphenyleneiodonium chloride (DPI), suggesting a role for NOX in this pathway. Indeed, in a swine model of ischemia reperfusion injury, NOX-dependent superoxide production was inhibited by a C1 inhibitor, recombinant C1-INH, with a concomitant reduction in NOX4 and NOX2 protein expression in kidney homogenates (132).

Using human proximal tubular cells, C3a stimulated NOX-dependent superoxide production and alpha-smooth muscle actin (α-SMA) expression (132). The C3a-stimulated increase in α-SMA expression, which is a specific marker for fibrotic myofibroblasts, was inhibited by NOX4 RNA silencing, suggesting that C3a mediates superoxide production and renal fibrosis through an NOX4-dependent pathway (132). In another study using mouse kidney endothelial cells, C5a treatment stimulated ROS production as measured by a fluorogenic probe, 2′,7′-dichlorofluorescin diacetate, that on oxidation, measures ROS production within the cell (150).

Further, C5a-induced ROS generation was diminished in the presence of a pan NOX inhibitor, VAS2979, which demonstrated that C5a triggered oxidative stress via NOX-dependent ROS generation (150). Collectively, these findings point toward an important role for complement end products in mediating oxidative stress in renal cells, although not in the context of hyperglycemia or diabetes. Indeed, this review has presented evidence that complement end products such as MAC and the anaphylatoxins C3a and C5a are activated in diabetes, with NOX4 as a key source of ROS responsible for renal injury in diabetes (71); it would not be surprising if the complement probably plays a role in promoting oxidative stress and renal injury in DKD through the actions of NOX isoforms.

## Targeting the Complement Pathway in DKD

To date, very few complement targeting treatments have been approved for use in humans, primarily because of the increased risk of infection. The most well known is eculizumab, which has been successful clinically and approved for paroxysmal nocturnal hemoglobinuria (PNH) and atypical hemolytic uremic syndrome (aHUS) (116). Eculizumab (tradename Soliris) is a long-acting humanized anti-C5 monoclonal antibody (mAb) that blocks the cleavage of C5 into C5a and C5b, thus inhibiting any downstream activation of the complement cascades, including C5a-C5aR1 signaling and MAC formation (34).

Patients are vaccinated against *Neisseria meningitides* before treatment with eculizumab, since blockade of the terminal complement system increases their susceptibility to meningococcal infection and other infections such as urinary, respiratory, and gastrointestinal tracts infection (34). Further, Eculizumab is once listed as the world's most expensive drug by Forbes, making it inaccessible to many. Since then, many more complement therapeutics are being investigated in clinical trials as potential treatment for PNH and aHUS, and approved drugs such as eculizumab have been tested as potential therapy for new indications (174).

Newer complement inhibitors currently being investigated in clinical trials for glomerular diseases are summarized in a recent review (174). However, no complement targeting therapies are currently under consideration for human DKD.

### Inhibition of anaphylatoxin receptors, C3aR and C5aR1

More recently, other complement targeted therapy that aimed at inhibiting more downstream effectors of the complement cascades have gained considerable ground, for example inhibitors of the anaphylatoxin receptors, C3aR and C5aR1 (60). Advantages of targeting the anaphylatoxin receptors include the availability of pharmacological antagonists, especially those targeting C5aR1; and the preservation of the immune defense function of the proximal complement cascades, leading to the production of opsonin C3b and terminal MAC.

A C3aR antagonist, SB290157 was shown to attenuate albuminuria and improve renal fibrosis in a rat model of T2D, as well as to inhibit inflammatory and fibrotic markers in human renal glomerular endothelial cells cultured in high-glucose conditions (85, 87). Inhibition of C3aR with SB290157 also preserved podocyte loss and normalized mitochondrial abnormalities in podocytes, including *Atp5i* and *Sod2* expression, which affects mitochondrial ATP and ROS production, respectively, in BTBR *ob*/*ob* mice (104). However, the nonspecific C3aR antagonist, SB290157, has been shown to have agonist activity on C3aR in a variety of cell systems (101) and has partial agonist activity on C5aR2 (92), thus making the interpretation of the role for C3aR in these studies uncertain.

Earlier studies exploring the role of C5a/C5aR1 in the pathogenesis of DKD employed the use of nonspecific C5a/C5aR inhibitors. K-76 sodium monocarboxylic acid (K-76 COONa), which is a nonspecific inhibitor of C5 and Factor I, has been shown to reduce albuminuria in a model of T2D, the Otsuka Long-Evans Tokushima Fatty (OLETF) rat (46). However, with the availability of more specific inhibitors, the role of C5aR1 in DKD is increasingly evident.

Yiu *et al.* showed that the inhibition of C5a using a mixed L-RNA/DNA Spiegelmer, NOX-D21, in *db/db* mice for 12 weeks attenuated glomerulosclerosis and tubulointerstitial damage; however, albuminuria and infiltration of macrophages into the kidney were not affected by the treatment (164). That study also showed that the inhibition of C5a with NOX-D21 reduced adipose differentiation-related protein (ADRP) in the diabetic kidney as well as the expression of *Dgat1* and *Srebp-1*, suggesting a role for C5a/C5aR1 in renal lipid metabolism (164).

Further, using a highly specific C5aR1 antagonist, PMX53, our group has shown that the inhibition of C5aR1 attenuated albuminuria, glomerulosclerosis, and tubulointerstitial fibrosis, as well as restoration of the loss of anti-inflammatory Tregs in C57BL/6J mice after 20 weeks of streptozotocin-induced diabetes (140).

**FIG. 6.**
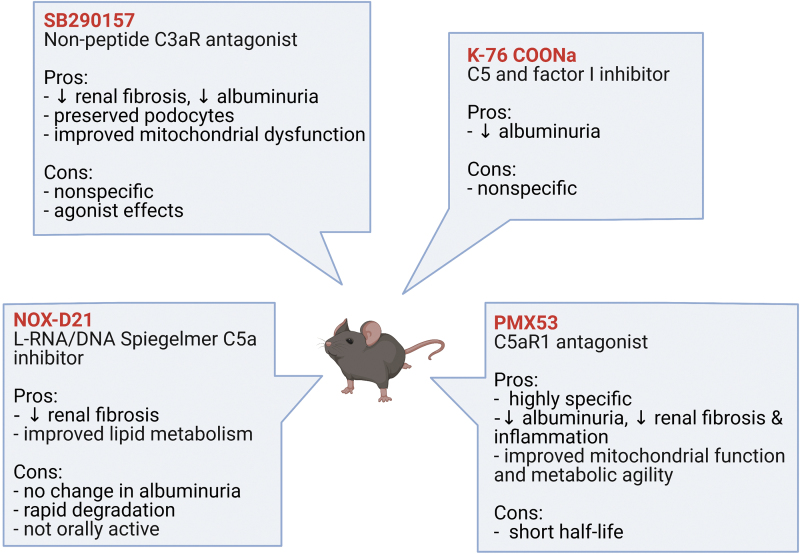
**Anaphylatoxin receptor inhibitors in preclinical studies of diabetic kidney disease.** A summary of preclinical studies that have shown promise using anaphylatoxin receptor inhibitors in rodent models of type 1 and type 2 diabetes, each with their advantages and disadvantages. Color images are available online.

These preclinical studies (summarized in Fig. 6), together with evidence from the human studies previously described, further strengthen the notion that C5a/C5aR1 signaling may be a key mediator of DKD.

Nonetheless, despite promising results from preclinical studies, clinical trials using anaphylatoxin receptor inhibitors have proved unsuccessful thus far (60). However, with many promising preclinical studies showing renoprotective effects from C3aR and C5aR1 inhibitors in DKD, complement targeted therapeutics in human DKD warrants further investigation.

## Conclusion

As is clear from the present review, a large body of evidence indicates the involvement of the complement cascade in the pathogenesis of DKD. The lectin pathway of complement activation is the most likely activator of the cascade in diabetes, presumably by autoreactivity toward altered self-surfaces known as DAMPs, including altered carbohydrate and acetylation patterns.

The mechanistic basis for the effects of complement activation in diabetic complications, however, remains incompletely elucidated, but unsurprisingly the major effector molecules of complement activation, the anaphylatoxins C3a and C5a and their interaction with their receptors as well as the terminal product of the complement cascade, the pore-forming membrane-attack complex are the most likely mediators. It appears that both C3a and C5a are likely to be key molecules that promote ROS generation and regulate immunometabolic pathways in the kidney, and both are suitable targets of therapy.

Since the currently used clinical therapies for DKD do not dampen the complement, future studies should aim at focusing on the development of novel antagonists that target the complement pathway to alleviate inflammation and kidney fibrosis, thereby reducing the burden of DKD, the major cause of ESRD worldwide.

## Abbreviations Used

α-SMA: alpha-smooth muscle actin
ADRP: adipose differentiation-related protein
AGEs: advanced glycation end products
aHUS: atypical hemolytic uremic syndrome
ASC: apoptosis-associated speck-like protein containing a caspase recruitment domain
ATP: adenosine triphosphate
C3aR: complement C3a receptor
C5aR1: complement C5a receptor 1
C5aR2: complement C5a receptor 2
CML: N-ɛ-Carboxymethyllysine
CR: complement receptor
CTGF: connective tissue growth factor
DAF: decay accelerating factor
DAMPs: danger-associated molecular patterns
DEGs: differentially expressed genes
DKD: diabetic kidney disease
DPI: diphenyleneiodonium chloride
DSS: dextran sulfate sodium
E-CR1: erythrocyte CR1
eGFR: estimated GFR
ERK: extracellular signal-regulated kinase
ESRD: end stage renal disease
FADH_2_: flavin adenine dinucleotide
FAO: fatty acid β-oxidation
FB: factor B
FCCP: trifluoromethoxy carbonylcyanide phenylhydrazone
FD: factor D
GFR: glomerular filtration rate
GLP-2: glucagon-like peptide-2
hsCRP: high-sensitive C-reactive protein
IL: interleukin
KEGG: Kyoto Encyclopedia of Genes and Genomes
KIM-1: kidney injury molecule
LPS: lipopolysaccharide
mAb: monoclonal antibody
MAC: membrane attack complex
Map19: mannose binding lectin-associated protein of 19
MBL: mannose-binding lectin
MCP-1: monocyte chemoattractant protein-1
NADH: nicotinamide adenine dinucleotide
NDRD: nondiabetic renal disease
NF-κB: nuclear factor kappa B
NLRs: NOD-like receptors
NLRP3: NOD-, leucine-rich repeats- and pyrin domain-containing 3
NOD: nucleotide-binding oligomerization domain
NOX: nicotinamide adenine dinucleotide phosphate oxidase
OLETF: Otsuka Long-Evans Tokushima Fatty
OXPHOS: oxidative phosphorylation
PAMPs: pathogen-associated molecular patterns
PDGF: platelet-derived growth factor
PKC: protein kinase C
PKR: protein kinase R
PNH: paroxysmal nocturnal hemoglobinuria
PPAR: peroxisome proliferator-activated receptor
PRM: pattern recognition molecules
RAS: renin angiotensin system
ROS: reactive oxygen species
SGLT2: sodium glucose cotransporter 2
SMC: smooth muscle cells
SOD2: superoxide dismutase 2
T1D: type 1 diabetes
T2D: type 2 diabetes
TCA: tricarboxylic acid
TGF-β: transforming growth factor-β
TLR4: toll like receptor 4
TNF: tumor necrosis factor
TNFR: tumor necrosis factor receptor
Treg: regulatory T cells
VDAC: voltage-dependent anion channels
WT: wildtype
ZS: Zucker fatty diabetic/spontaneous hypertensive heart failure
